# Cervical Cytopathological Findings in Korean Women with *Chlamydia trachomatis*, *Mycoplasma hominis*, and *Ureaplasma urealyticum* Infections

**DOI:** 10.1155/2014/756713

**Published:** 2014-01-08

**Authors:** Yuri Choi, Jaesook Roh

**Affiliations:** Laboratory of Reproductive Endocrinology, Department of Anatomy & Cell Biology, College of Medicine, Hanyang University, San 17 Haengdang-dong, Seongdong-gu, Seoul 133-792, Republic of Korea

## Abstract

This is to investigate the cervical cytological abnormalities associated with *Chlamydia trachomatis*, *Mycoplasma hominis*, *Mycoplasma genitalium,* and *Ureaplasma urealyticum* infections on routine screen. A total of 714 subjects who had undergone cervical Pap smears and concomitant analyses for cervical infections were included by a retrospective search. The frequencies of reactive cellular change (RCC) and squamous epithelial abnormalities were significantly higher in *Chlamydia* positive subjects than in uninfected subjects (*P* < 0.001). Of the 124 subjects tested for *M. hominis*, *M. genitalium*, and *U. urealyticum*, 14 (11%) were positive for *M. hominis* and 29 (23%) were positive for *U. urealyticum*. Squamous abnormalities were more frequent in subjects with *Ureaplasma* infections than in uninfected subjects (24% versus 8%). Taking together these findings, *C. trachomatis* and *U. urealyticum* may have a causal role in the development of cervical epithelial changes, including RCC. Thus, extra awareness is warranted in cervical screening of women with *Chlamydia* or *Ureaplasma* infections.

## 1. Introduction

It is relatively common for cervical smear results to show reactive cellular changes (RCC); these are classified as presence of nuclear enlargement, finely and evenly distributed chromatin, absence of hyperchromasia, smooth nuclear outline, and no significant increase in nuclear/cytoplasmic ratio [1]. Histological indicators of RCC according to the Bethesda System 3 classification [1, 2] include nonneoplastic morphologic changes associated with inflammation, radiation effects, intrauterine devices, and atrophy. Thus, RCC is often related to trauma, hormonal changes, and sexually transmitted pathogens, amongst other factors. We usually overlook RCC on Pap smears as RCC was shown to be within normal limits after restricting atypia to the category of atypical squamous cells of undetermined significance (ASCUS) [1, 2]. However, several studies have shown an increased frequency of squamous lesions in women with RCC compared to women with negative smears, although the frequencies differed between studies [3–5]. A causal relationship has been suggested between epithelial lesions and some sexually transmitted infections [6–10]. Some studies have shown cervical changes (mainly inflammatory) in populations with microbiologically confirmed infections regardless of whether symptoms were present or not [9, 10]. Furthermore, some sexually transmitted agents have been considered as possible cofactors in the pathogenesis of epithelial lesions such as ASCUS and squamous intraepithelial lesion (SIL), although no single agent has been identified as particularly significant [5, 8]. Taken together these findings suggest that RCC may require attention, especially in cases involving sexually transmitted infections. Therefore, the objective of this study was to assess the relationship between Pap smear abnormalities and cervicovaginal infection. For this purpose, we compared the number and type of cytological abnormalities on routinely examined Pap smears of the uterine cervix in women with or without *Chlamydia*, *Mycoplasma*, or *Ureaplasma* infections.

## 2. Methods

We retrospectively reviewed all women attending general gynecological outpatient clinics for prepregnancy check-ups who underwent routine screening with Pap smears and concomitant microbiological analyses for cervical infections at Hanyang University Medical Center between April 2008 and December 2012. The study was approved by the hospital's Institutional Review Board for Clinical Research (HYUHIRB-2009-R-50). No patient identifiers were disclosed. Cases were identified by an electronic database search for test results for *Chlamydia, Mycoplasma*, or *Ureaplasma* screening and cervicovaginal Pap smears in accordance with the 2001 Bethesda System [2]. All subjects were sexually active and aged less than 45. The ages of the subjects ranged from 20 to 45 years (mean 38.2 years). Exclusion criteria were any type of cancer, abnormal Pap smears during the previous year, any known or suspected immunodeficiency, and chronic disease. Any patients infected with more than two organisms were also excluded, because combined infections may have confounding effects on cellular changes. In total 714 subjects were included in the study. All had undergone microbiological testing for *C. trachomatis,* and 124 had also undergone testing for *M. hominis*, *M. genitalium,* and *U. urealyticum*. All Pap smears were liquid-based preparations and *Chlamydia*, *Mycoplasma,* and *Ureaplasma* DNAs were detected by polymerase chain reaction assay. Briefly, genomic DNA was extracted from the cervical samples using the QIAamp DNA miniKit (Qiagen, Crawley, UK) according to the manufacturer's instructions. Polymerase chain reaction was performed using commercial premix real-time PCR kits (AccuPower real-time PCR kit) (Bioneer, Daejeon, Korea).

We reviewed Pap smear results and concomitant microbiological test results from clinical records and investigated associations between infections and abnormal Pap smears. Analysis variables were age, parity, number of abortions, presence of nonspecific vaginal discharge or urethritis, Pap smear result, and cervical infection. In the cytopathological analysis, normal findings are presented as negative results. Abnormal epithelial changes are classified as ASCUS, low grade squamous intraepithelial lesion (LSIL), or high grade squamous intraepithelial lesion (HSIL). For convenience, we defined RCC as changes that did not fit well-established criteria for condyloma or dysplasia.

All data are presented as mean ± SD (standard deviation of the mean). Data were analyzed with Student's *t*-test or chi-square test, using SPSS 17.0 for Windows (SPSS Inc., Chicago, IL). A *P* value < 0.05 was taken to indicate significance.

## 3. Results

The data are summarized in Table 1. Nineteen women (3%) had positive PCR results for *C. trachomatis*. Comparison of *Chlamydia*-infected and uninfected subjects showed that the mean age of *Chlamydia* positive subjects was lower than that of uninfected subjects (27.7 years versus 39.2 years). The mean number of previous spontaneous abortions was lower in *Chlamydia *positive subjects than in uninfected subjects (0.2 versus 0.9). No significant differences between infected and uninfected subjects were found for number of births and presence of nonspecific symptoms. A total of 695 women (97%) were negative for *Chlamydia*. The Pap smear results for these subjects were as follows: 478 (69%) were classified as having negative smears, 175 (25%) were diagnosed with RCC, 15 (2.2%) were diagnosed with ASCUS, 20 (2.9%) were diagnosed with LSIL, and 7 were diagnosed with HSIL (1.0%). Thus, of the 695 women in the *Chlamydia* negative group, 42 (6%) had significant Pap smear abnormalities. Of the 19 *Chlamydia* positive cases, 12 (63%) had RCC and 2 (11%) had some degree of abnormal cytology (either ASCUS or LSIL). The frequencies of RCC and squamous abnormalities were significantly higher in *Chlamydia* positive subjects than in uninfected subjects (63% versus 25% and 11% versus 6%, resp.; *P* < 0.001 for both).

Of the 124 subjects tested for *M. hominis*, *M. genitalium*, and *U. urealyticum*, 14 (11%) were positive for *M. hominis*, 29 (23%) were positive for *U. urealyticum*, and no *M. genitalium* was detected. Cytopathological analysis revealed negative results in 72 (58%) cases, RCC in 32 cases (30%), and squamous abnormalities in 15 cases (12%), including ASCUS, LSIL, and HSIL. Age, parity, number of previous spontaneous abortions, and number of cases with nonspecific symptoms were similar in uninfected subjects and subjects infected with *M. hominis* or *U. urealyticum*.

In cases with *M. hominis* infection, the frequencies of RCC and LSIL were both 14% (2/14). In *M. hominis* negative cases, the frequency of RCC was 32% (35/110) and the frequency of squamous abnormalities was 12% (13/110; 5 ASCUS, 7 LSIL, and 1 HSIL). The frequencies of RCC and squamous abnormalities were not significantly different between infected and uninfected subjects (*P* = 0.399).

Of the 29 *Ureaplasma* positive cases, 13 (45%) had normal cytology, 9 (31%) had RCC, and 7 (24%) had squamous abnormalities (2 ASCUS, 5 LSIL). Unexpectedly, squamous abnormalities were more frequent in subjects with *Ureaplasma *infections than in uninfected subjects (24% versus 8%), although the difference was not statistically significant (*P* = 0.054).

## 4. Discussion

RCC is a common finding in cervical Pap smears. The prevalence of RCC may vary depending on various epidemiological factors. Frequencies ranging from 22% to 80% have been reported [11–13] (consistent with our figure of 26% in routine Pap smear). The clinical significance of RCC on Pap smears from asymptomatic women is still controversial and no consensus exists on the management of RCC. Since a higher prevalence of squamous abnormalities has been found in women with dysbacteriosis [6], we assessed the possible association between cellular changes and the presence of cervical pathogens in this study. We found a higher frequency of RCC in *Chlamydia* positive women, but not in *M. hominis* or *U. urealyticum* positive women. *U. urealyticum* may however have a causal role in the development of squamous abnormalities, although the effect seen was not statistically significant (*P* = 0.054) probably because of the small number of study subjects; further studies are required.

*C. trachomatis* is the most common bacterial sexually transmitted infection worldwide [14]. The prevalence of *Chlamydia* infections in routine screening of women has been reported to be 2–10% [15–19], with the figure varying according to epidemiological factors and methodological differences. A higher incidence was reported in women less than 35 years of age and is often associated with unhealthy lifestyle factors [18]. Consistent with these findings, *Chlamydia* was detected in 3% of the subjects in this study, and these subjects were significantly younger than the uninfected subjects. *Chlamydia* infection can cause a variety of upper and lower genitourinary symptoms including nonspecific vaginal discharge or urinary symptoms, but most infected patients are asymptomatic, as in this study. *Chlamydia* infection is also known to be a risk factor for spontaneous abortion [16], but we found histories of previous spontaneous abortions were much fewer in *Chlamydia* positive subjects than uninfected subjects. This conflicting result could be explained by the differences in age and parity between the two groups.

The association between *Chlamydia* and abnormal cytology has previously been examined with various methodologies and populations. The role of *Chlamydia* in the pathogenesis of squamous lesions remains unknown, but large epidemiological studies suggest that *Chlamydia* infection may be associated with abnormal Pap smears [18, 20, 21]. Associations with both early stages of lesions [15, 20, 21] and more advanced stages [18, 22] have been reported. In addition, chronic *Chlamydia* infection is associated with cervical cellular hypertrophy, mild atypia [23], and even invasive cancer [18]. We found that the frequency of cellular changes, including RCC, was significantly greater in *Chlamydia* positive women than in uninfected women (74% versus 31%), consistent with previous reports. However, there are several studies showing no association or even a negative correlation between *Chlamydia* infection and the development of cervical lesions [16, 17, 23]. Thus, the role of *Chlamydia* infection as a risk factor in the development of cervical lesions is still controversial.

Although RCC is classified as being within normal limits, it may be associated with more advanced lesions and it is worth considering whether there is any possible association between *Chlamydia* and RCC. *Chlamydia* infection has been reported to be associated with RCC [7, 24], with more than 80% of *Chlamydia* positive cases showing RCC in one study [24]. This is similar to our finding that 63% of the subjects positive for *Chlamydia* showed RCC, compared to 25% of *Chlamydia* negative women. Although the small sample size and the fact that residual confounding by unknown factors could not be eliminated are limitations of our study, our results suggest that *Chlamydia* infection may contribute to the development of cervical cellular changes, including reactive changes.

*Mycoplasmas* are also commonly present in the genital tract of sexually active women, in particular *M. hominis* and *U. urealyticum*. Previous studies have reported a colonization rate between 10% and 50% for *U. urealyticum*, but less than 30% for *M. hominis* [25, 26]. The higher prevalence of *U. urealyticum* is consistent with our results, although we found lower rates of both *M. hominis* (11%) and *U. urealyticum* (23%) colonization than in other reports. This may be due to differences in the study populations, since we only selected women who had undergone routine screening and excluded women with simultaneous colonization with multiple organisms. It has been hypothesized that *M. hominis* and *U. urealyticum* play a role in cervical cytological pathogenesis and increased risk of HPV infection, and women with cervical cytological abnormalities have been found to present with high frequencies of *M. hominis* and *Ureaplasma* infections [27–29]. Consistent with this, we found a higher incidence of SIL in *Ureaplasma* positive women than in *Ureaplasma* negative women (24% versus 8%); HPV DNA data were not available to us. The role of *Mycoplasma* or *Ureaplasma* as primary causative agents of cytological cervical lesions is still controversial, but they are recognized as initiating factors in cervical inflammation [29] and therefore may be involved in early cervical cellular changes including RCC. However, we did not find any statistically significant differences in the frequencies of RCC between uninfected women and women infected with *M. hominis* or *Ureaplasma*. This could be due to the small sample size; hence further studies are required.

Human genital epithelial cells have also been found to be susceptible to *M. genitalium* infection [30]. However, the prevalence of *M. genitalium* can be less than 1% [31], and there were no positive cases in this study. Thus the role of *M. genitalium* as an etiological agent of RCC remains to be clarified.

*C. trachomatis* is not only responsible for inflammation in the upper genital tract and for pelvic inflammatory disease but is also likely to be one of the causes of cervical epithelial changes. *U. urealyticum* may also have a causal role in the development of squamous abnormalities, although our results were not statistically significant. There were only a few subjects with infections in this study and the question of the existence of epithelial lesions in the subgroup with RCC is still controversial, but nonetheless we would recommend being on the lookout for *Chlamydia* or *Ureaplasma* infected cells with epithelial cell changes, including RCC. Our findings may help clinicians decide whether additional microbiological testing of women presenting with RCC is indicated, and they show that extra awareness is warranted in cervical screening of women with *Chlamydia* or *Ureaplasma* infections.

## Figures and Tables

**Table 1:** Comparison of the general clinical characteristics and cytopathological results from preconception screening of Korean women with and without *Chlamydia trachomatis*, *Mycoplasma hominis,* and *Ureaplasma urealyticum* infections.

	*Chlamydia trachomatis *		*P *value	*Mycoplasma hominis *		*P* value	*Ureaplasma urealyticum *		*P* value
	(+)	(−)		(+)	(−)		(+)	(−)	
No. of cases (%)	19 (3)	695 (97)		14 (11)	110 (89)		29 (23)	95 (77)	
Mean age	27.7 ± 6.1	39.2 ± 5.9	<.001	39.6 ± 5.5	37.8 ± 6.6	0.566	38.1 ± 9.6	37.9 ± 7.1	0.937
Parity	0.9 ± 0.9	1.4 ± 1.2	0.081	1.2 ± 0.9	1.1 ± 1.0	0.986	1.0 ± 1.1	1.2 ± 1.0	0.321
Previous number of SA	0.2 ± 0.4	0.9 ± 1.4	<.001	1.0 ± 1.6	0.9 ± 1.2	0.687	1.0 ± 1.4	0.9 ± 1.3	0.883
Presence of symptoms	5 (26)	206 (30)	0.754	6 (43)	26 (21)	0.117	8 (28)	24 (25)	0.802
Pap smear			<.001			0.399			0.054
Negative	5 (26)	478 (69)		10 (71)	62 (56)		13 (45)	59 (62)	
RCC	12 (63)	175 (25)	<.001	2 (14)	35 (32)	0.149	9 (31)	28 (29)	0.298
Abnormal	2 (11)	42 (6)		2 (14)	13 (12)		7 (24)	8 (8)	
ASCUS	1	15		—	5		2	3	
LSIL	1	20		2	7		5	4	
HSIL	—	7		—	1		—	1	

Values are represented as number (%) and mean ± SD. (+): PCR positive; (−): PCR negative; SA: spontaneous abortion; RCC: reactive cellular change; ASCUS: atypical squamous cell-undetermined significance; LSIL: low grade squamous intraepithelial lesion; HSIL: high grade squamous intraepithelial lesion. *P* values are for comparisons between cases with positive and negative microbiological test results.
